# Care Settings, Autonomy, and Costs in Severe Mental Illness: A Cross-Sectional Comparison of Hospital, Residential, and Community Care in Romania

**DOI:** 10.3390/healthcare14070884

**Published:** 2026-03-30

**Authors:** Elena Tanase, Ion Radu, Adrian Cosmin Ilie, Dan-Alexandru Surducan, Adina Bucur, Alina Tischer, Felicia Marc, Ion Papava, Sorin Ursoniu

**Affiliations:** 1 Doctoral School, Faculty of Medicine, “Victor Babes” University of Medicine and Pharmacy Timisoara, 300041 Timisoara, Romania; elena.tanase@umft.ro; 2 Department of Functional Sciences, Discipline of Public Health, Center for Translational Research and Systems Medicine, “Victor Babes” University of Medicine and Pharmacy Timisoara, 300041 Timisoara, Romania; radu.ion@umft.ro (I.R.); ilie.adrian@umft.ro (A.C.I.); surducan.dan@umft.ro (D.-A.S.); bucur.adina@umft.ro (A.B.); sursoniu@umft.ro (S.U.); 3 Department of Otorhinolaryngology, “Victor Babes” University of Medicine and Pharmacy Timisoara, 300041 Timisoara, Romania; 4 Department of Medical Sciences, Faculty of Medicine and Pharmacy, University of Oradea, 410073 Oradea, Romania; 5 Department of Neurosciences-Psychiatry, Faculty of Medicine and Pharmacy, University of Oradea, 410073 Oradea, Romania; papava.ion@umft.ro

**Keywords:** severe mental illness, deinstitutionalization, community mental health services, quality of life, health expenditures

## Abstract

**Background and Objectives**: Romania’s transition from long-stay psychiatric care to more balanced community-oriented care remains incomplete, and comparative patient-level data are limited. The primary objective was to compare mental health-related quality of life, autonomy, perceived coercion, and direct mental health costs across hospital, residential, and community care settings. Secondary objectives were (i) to compare hospital care with deinstitutionalized care taken together (residential + community), and (ii) to examine whether autonomy and perceived coercion were associated with mental health-related quality of life. **Methods**: In this cross-sectional study, 128 adults with severe mental illness (42 hospital, 43 residential, 43 community) completed the Romanian SF-36 v2.0 and two brief study-specific rating scales for autonomy and perceived coercion. Service-use and cost data for the previous 12 months were extracted from records. **Results**: Community participants had higher SF-36 mental scores than hospital patients (63.3 ± 7.6 vs. 52.8 ± 9.1) and higher autonomy (72.1 ± 10.0 vs. 53.0 ± 8.6), with lower perceived coercion (4.2 ± 1.3 vs. 6.4 ± 1.5; all *p* < 0.001). Mean combined direct costs in residential and community settings were approximately USD 1000 lower than in hospital care (USD 2671.6 vs. 3666.2; *p* < 0.001). When residential and community participants were analyzed together as a deinstitutionalized group, they also had higher SF-36 mental scores (59.7 ± 8.9 vs. 52.8 ± 9.1; *p* < 0.001). In multivariable models (R^2^ = 0.316), each 10-point higher autonomy score was associated with a 2.8-point higher SF-36 mental score, whereas each 1-point higher coercion score was associated with a 1.3-point lower score. Exploratory mediation analysis suggested that autonomy statistically attenuated the association between deinstitutionalized care and mental quality of life. **Conclusions**: In this sample, residential and community arrangements outside hospital wards were associated with better mental health-related quality of life, higher autonomy, lower perceived coercion, and lower direct costs than long-stay hospital care. These findings support the study objective of comparing Romanian care settings and suggest that autonomy is an important correlate to target in future service reconfiguration and longitudinal research.

## 1. Introduction

Deinstitutionalization has reshaped mental health systems worldwide over the past six decades, but the balanced care model emphasizes that hospital and community services should be developed as a coordinated continuum rather than as mutually exclusive alternatives [1,2,3,4,5]. In Central and Eastern Europe, this rebalancing has progressed unevenly, with many systems still relying on long-stay beds and only partially developed community supports [1]. In Romania, the resulting landscape includes psychiatric hospitals, residential social care homes, and a smaller but growing sector of community protected housing and community mental health services [6,7,8,9].

Within this continuum, residential care is conceptually intermediate between hospital wards and more community-integrated protected housing [10,11,12,13,14,15]. International studies of supported accommodation show that these settings differ not only in staffing intensity and supervision, but also in autonomy, service quality, and costs [16]. This distinction is important in Romania, where not all arrangements labeled as community-based correspond to fully independent private living; in our setting, community care refers to small shared apartments with structured community follow-up.

Health-related quality of life, perceived autonomy, and perceived coercion are especially relevant outcomes when evaluating long-term mental health care. Recovery-oriented frameworks emphasize self-determination, voice, and everyday participation, while research on coercion shows that subjective pressure can persist even outside formally involuntary admissions [3,4,10,11,12,13,14]. Romanian evidence remains limited and has focused mainly on general quality of life among long-term service users rather than direct comparisons across hospital, residential, and community arrangements [17].

Economic comparisons are also needed. Severe mental illness is associated with high direct and indirect costs, and prolonged institutional care is resource intensive [18,19,20,21,22,23,24]. At the same time, residential and community services shift spending toward staff support, rehabilitation, and social care rather than eliminating costs altogether [1,16]. Comparative outcome and cost data are therefore essential if deinstitutionalization is to be evaluated as a recovery-oriented strategy rather than only as a budgetary reform.

Against this background, the primary objective of this study was to compare three Romanian care settings—psychiatric hospital care, long-term residential homes, and community protected housing—with regard to health-related quality of life, autonomy, perceived coercion, and direct mental health costs. The residential setting was conceptualized a priori as an intermediate form of support between hospital care and community protected housing.

Secondary objectives were to compare hospital care with deinstitutionalized arrangements taken together (residential + community) and to examine which socio-demographic, clinical, and experiential factors were associated with better mental health-related quality of life. We hypothesized that the most community-integrated arrangement (protected housing) would show the most favorable profile, residential care would occupy an intermediate position, and combined deinstitutionalized care would be associated with equal or lower direct costs than long-stay hospital care.

## 2. Materials and Methods

### 2.1. Study Design and Setting

We conducted a cross-sectional observational study between October 2023 and October 2025 in Timiș County, Western Romania. A cross-sectional design was chosen because the immediate aim was to compare contemporaneous patient-reported outcomes and recent service-use costs across existing care arrangements under routine conditions; a harmonized longitudinal registry linking health and social care sectors was not available, and many service transitions had occurred before study initiation. Participants were recruited from three service types providing ongoing care for adults with severe mental illness (SMI): (1) two psychiatric hospitals, including long-stay wards; (2) two long-term residential homes; and (3) community mental health services offering protected apartments and intensive outpatient follow-up. The study was approved by the Institutional Review Board of “Victor Babeș” University of Medicine and Pharmacy Timisoara (Approval no. 94, 2 October 2023) and by each participating institution. All procedures followed the Declaration of Helsinki and national regulations regarding research in vulnerable populations.

These settings were conceptualized as a graded continuum of institutional support rather than as identical forms of community living. Hospitals provided acute and extended inpatient care with 24 h medical staffing. Residential homes provided long-term accommodation with nursing and social care staff but no on-site psychiatrists. Community protected housing consisted of small shared apartments, not independent private tenancies, with residents supported by multidisciplinary community mental health teams and family physicians. All sites were publicly funded through the National Health Insurance House and local social care budgets.

### 2.2. Participants and Recruitment

Eligible participants were adults aged 18–70 years with a documented DSM-5 diagnosis of schizophrenia spectrum disorder, bipolar disorder, major depressive disorder with psychotic features, or schizoaffective disorder. Additional inclusion criteria were: (1) at least 12 months of continuous contact with the current service (hospital, residential home, or community program), and (2) clinical stability sufficient to complete interviews, as judged by the treating psychiatrist or responsible physician. We excluded individuals with severe intellectual disability, advanced neurocognitive disorders, acute intoxication or withdrawal, and those under compulsory forensic measures at the time of recruitment. Placement in hospital, residential, or community care was determined before study enrolment through routine clinical and social care decision-making based on illness course, functional status, family support, and local service availability; no participant was allocated to a setting by the study.

Consecutive sampling was used at each site until 128 participants were enrolled (42 hospital, 43 residential, and 43 community). The target sample size was planned to provide approximately 80% power at α = 0.05 to detect a medium between-group effect in a three-group comparison (ANOVA f ≈ 0.28), while remaining feasible within the number of eligible service users available during the recruitment period. Near-balanced group sizes were chosen to support both three-setting comparisons and prespecified hospital versus deinstitutionalized analyses.

### 2.3. Measures and Data Collection

Socio-demographic and clinical data included age, sex, years of education, employment status, monthly income category (coded as low income for personal monthly income below 3600 Romanian lei [RON], approximately <USD 800 during the analytic period), primary psychiatric diagnosis, illness duration, number of prior psychiatric hospitalizations, and years in the current setting. Diagnoses were extracted from medical records and coded according to DSM-5 categories. We also recorded indicators of social support, including at least weekly face-to-face or telephone contact with family or close friends, and participation in structured day programs (ergotherapy, occupational therapy, or psychoeducation) at least once per week.

Health-related quality of life was assessed using the Romanian version of the Short Form-36 Health Survey, version 2.0 (SF-36 v2.0), for which Romanian translation, population norms, and psychometric evaluation have been previously reported [25,26]. We calculated the Physical Component Summary (PCS) and Mental Component Summary (MCS) scores on a 0–100 scale, with higher scores indicating better health status. Autonomy was measured using a brief study-specific 0–100 visual analog item anchored at “no influence over my daily life and treatment” and “full influence over my daily life and treatment”. Perceived coercion was assessed using a study-specific 0–10 numeric rating item reflecting the extent to which participants felt pressured or constrained by mental health services in the last year. These two brief items were developed ad hoc for this study because no short Romanian routine-care instrument covering both constructs across all three settings was available; they were piloted in 12 patients for wording clarity and face validity. Because each was a single-item measure, internal-consistency coefficients were not applicable.

Service-use and cost data were extracted from hospital information systems, residential facility records, and community program logs. For each participant, we recorded the number of psychiatric inpatient days and specialist outpatient visits in the preceding 12 months. Direct mental health costs included inpatient bed-day tariffs, outpatient visit tariffs, psychotropic medication expenditures, and community support costs, the latter comprising case-management contacts, psychiatrist/psychologist/nurse visits, social-work input, structured day programs, and supported-housing supervision. Costs were recorded in RON and are presented in RON with approximate USD equivalents for international comparability, using the mean National Bank of Romania USD/RON exchange rate for 2024 (approximately 1 USD = 4.6 RON) [27].

### 2.4. Statistical Analysis

All analyses were conducted using SPSS v28.0 (IBM Corp., Armonk, NY, USA) and double-checked in R 4.3. Continuous variables were examined for distributional assumptions using the Shapiro–Wilk test, histograms, Q–Q plots, and inspection of ANOVA residuals. Normally distributed variables are reported as mean ± standard deviation (SD); non-normal variables are summarized as median and interquartile range, although parametric comparisons were retained when residual diagnostics were acceptable. For variables showing mild right-skew, sensitivity analyses with non-parametric tests yielded the same direction of inference. Missing data were minimal (<3% for any variable) and were handled using complete-case analysis.

Group differences across the three settings (hospital, residential, community) were evaluated with one-way analysis of variance (ANOVA) for continuous variables and chi-square tests for categorical variables. When ANOVA results were significant, we explored pairwise differences using Tukey’s post hoc tests. Because residential and community settings both represent forms of care outside hospital wards within Romania’s deinstitutionalization process, we prespecified a secondary analytic contrast between hospital care and a combined deinstitutionalized group (residential + community), evaluated with independent-samples *t*-tests or chi-square tests as appropriate. Pearson correlation coefficients quantified associations between quality-of-life scores, autonomy, perceived coercion, and total costs.

To identify independent correlates of mental health-related quality of life, we fitted a multivariable linear regression model with SF-36 MCS as the dependent variable. Independent variables were selected a priori based on clinical plausibility and prior literature: age, sex, low-income status, psychotic versus non-psychotic diagnosis, deinstitutionalized versus hospital setting, autonomy score, and perceived coercion. Variables were entered simultaneously. We examined multicollinearity using variance inflation factors (VIF < 3 for all predictors), and model assumptions were checked through residual plots. Statistical significance was defined as a two-sided *p*-value ≤ 0.05. We report unstandardized coefficients to preserve direct interpretability in SF-36 points, together with R^2^ as a model-level effect size.

As exploratory secondary analyses, we fitted a negative binomial regression model for annual inpatient days to account for overdispersed count data, an ordinary least squares mediation model with non-parametric bootstrap confidence intervals to examine whether autonomy statistically attenuated the association between setting and MCS, and a latent profile analysis (LPA) to identify subgroups based on MCS, autonomy, coercion, service use, and costs. These analyses were considered hypothesis-generating because of the cross-sectional design and modest sample size.

## 3. Results

Table 1 summarizes socio-demographic and clinical characteristics across the three settings. The final sample comprised 42 hospital patients, 43 residents of long-term homes, and 43 individuals in community protected housing (*n* = 128). Mean age differed by setting, with residential participants older on average (53.8 ± 8.8 years) than hospital participants (47.7 ± 10.6 years) and community participants (45.1 ± 11.2 years; ANOVA *p* = 0.001). Years of education were 11.2 ± 1.9 in hospital care, 9.8 ± 2.0 in residential care, and 11.4 ± 2.1 in community housing (*p* < 0.001).

Women represented 54.8% of hospital participants, 58.1% of residential participants, and 37.2% of community participants (chi-square *p* = 0.11). Low-income status was common in all three groups (73.8%, 65.1%, and 58.1%, respectively; *p* = 0.31). Mean illness duration was 12.5 ± 5.2 years in hospitals, 14.1 ± 6.1 years in residential homes, and 10.7 ± 4.1 years in community housing (*p* = 0.015). Psychotic disorders were the most frequent diagnoses across all settings (45.2%, 62.8%, and 44.2%, respectively; *p* = 0.15).

Indicators of institutional trajectory and service configuration are shown in Table 2. Prior psychiatric hospitalizations averaged 7.4 ± 2.7 in hospital care, 5.6 ± 3.8 in residential care, and 4.4 ± 3.0 in community care (ANOVA *p* < 0.001). Years spent in the current setting were 5.1 ± 2.7, 7.1 ± 3.5, and 2.9 ± 1.9, respectively (*p* < 0.001).

Participation in structured day programs was 52.4% in hospital wards, 58.1% in residential homes, and 81.4% in community housing (chi-square *p* = 0.013). Weekly family contact was reported by 42.9%, 48.8%, and 67.4% of participants, respectively (*p* = 0.060).

Quality-of-life and experiential outcomes are detailed in Table 3. SF-36 physical component scores were 57.1 ± 8.7 in hospitals, 58.0 ± 7.9 in residential homes, and 61.9 ± 7.7 in community housing (ANOVA *p* = 0.016). SF-36 mental component scores were 52.8 ± 9.1, 56.0 ± 8.6, and 63.3 ± 7.6, respectively (*p* < 0.001). Post hoc comparisons showed that community participants scored higher than both hospital and residential groups. Mean autonomy scores were 53.0 ± 8.6, 57.7 ± 9.5, and 72.1 ± 10.0 (*p* < 0.001), while perceived coercion scores were 6.4 ± 1.5, 6.1 ± 1.6, and 4.2 ± 1.3 (*p* < 0.001).

Annualized service-use and cost data are presented in Table 4. Psychiatric inpatient days in the previous year averaged 47.5 ± 17.7 in hospital care, 17.0 ± 9.9 in residential care, and 7.8 ± 6.4 in community care (ANOVA *p* < 0.001). Outpatient specialist visits averaged 5.5 ± 2.7, 8.2 ± 3.3, and 12.4 ± 3.8, respectively (*p* < 0.001). Medication costs were similar across settings (around USD 1000 per year; *p* = 0.69). Community support costs—comprising staff support, day-program participation, and community follow-up—were higher in residential and community settings than in hospital care (USD 685.3 ± 169.5 and USD 957.8 ± 150.0 vs. USD 384.5 ± 113.5; *p* < 0.001). Total annual direct mental health costs per patient were USD 3666.2 ± 820.7 in hospitals, USD 2679.5 ± 480.8 in residential care, and USD 2663.8 ± 372.9 in community care (*p* < 0.001).

In the prespecified secondary comparison, the deinstitutionalized group was defined as the combined residential and community sample (*n* = 86). As shown in Table 5, readmission within 12 months occurred in 47.6% of hospital patients and 29.1% of deinstitutionalized participants (chi-square *p* = 0.062). Emergency department visits were reported in 40.5% and 31.4%, respectively (*p* = 0.41), and coercive measures were recorded in 26.2% and 19.8% (*p* = 0.55). Mean SF-36 mental component scores were 52.8 ± 9.1 in hospital care and 59.7 ± 8.9 in deinstitutionalized care (*t*-test *p* < 0.001). Mean total direct costs were USD 3666.2 ± 820.7 in hospital care and USD 2671.6 ± 427.8 in deinstitutionalized care (*p* < 0.001).

The multivariable linear regression model (Table 6) explained 31.6% of the variance in SF-36 mental scores (R^2^ = 0.316, F(7, 120) = 7.91, *p* < 0.001). After adjustment for socio-demographic characteristics and diagnosis, autonomy and perceived coercion remained independently associated with MCS. Each 10-point higher autonomy score was associated with a 2.79-point higher SF-36 mental score (SE = 0.70, *p* < 0.001), whereas each 1-point higher coercion score was associated with a 1.32-point lower score (SE = 0.48, *p* = 0.007). Age, sex, low-income status, psychotic diagnosis, and deinstitutionalized setting were not statistically significant at the 0.05 level.

**Figure 1:**
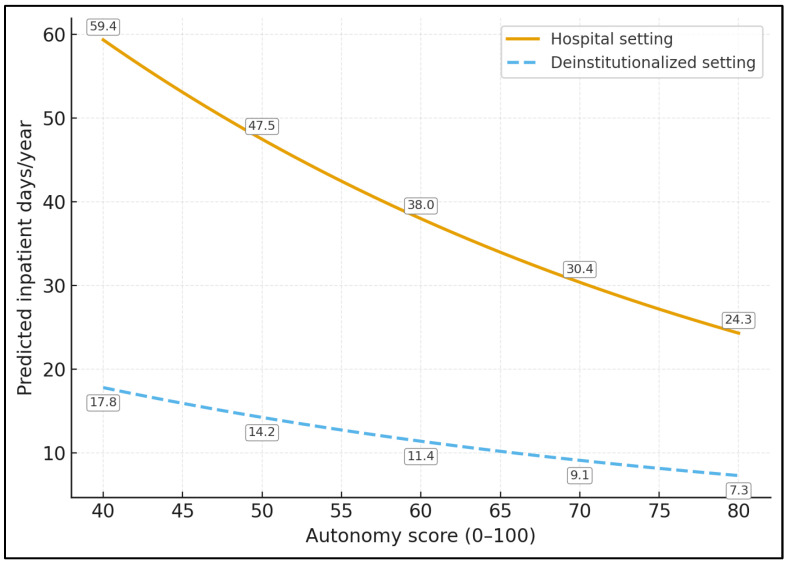
Predicted inpatient days by autonomy and care setting.

Figure 1 displays model-based predicted annual psychiatric inpatient days by autonomy score for hospital and deinstitutionalized settings using the negative binomial model from Table 7. Predicted inpatient use declined across higher autonomy values in both settings, with lower values across the full range for the deinstitutionalized group.

Table 7 presents the exploratory negative binomial regression for annual psychiatric inpatient days. Deinstitutionalized setting was associated with fewer inpatient days (IRR 0.3, 95% CI 0.2–0.4, *p* < 0.001). Higher autonomy was also associated with fewer inpatient days (IRR 0.8 per 10-point increase, 95% CI 0.7–0.9, *p* = 0.004), whereas higher perceived coercion was associated with more inpatient days (IRR 1.1 per 1-point increase, 95% CI 1.0–1.3, *p* = 0.043). Day-program participation was associated with fewer inpatient days (IRR 0.7, 95% CI 0.6–0.9, *p* = 0.006). Psychotic diagnosis and age were not significantly associated with the outcome.

These count-model results are presented as exploratory cross-sectional associations.

Table 8 summarizes an exploratory mediation analysis examining whether autonomy statistically attenuated the association between deinstitutionalized setting and SF-36 mental score. The total association between deinstitutionalized setting and mental score was 6.9 points (95% CI 3.5–10.3, *p* < 0.001). Deinstitutionalized setting was associated with an 11.4-point higher autonomy score (path a; 95% CI 7.9–14.9, *p* < 0.001), and autonomy was associated with higher SF-36 mental score after accounting for setting (path b = 0.3 per point, 95% CI 0.1–0.5, *p* = 0.003). The indirect effect was 3.5 points (bootstrapped 95% CI 1.6–5.4, *p* = 0.001), while the direct effect after including autonomy was 3.4 points (95% CI 0.3–6.5, *p* = 0.034). Given the cross-sectional design, this analysis should be interpreted as hypothesis-generating and not as evidence of causality.

Table 9 presents the exploratory latent profile analysis based on mental health-related quality of life, autonomy, perceived coercion, service use, and costs. Three profiles were identified: Class 1 (high-recovery; 35.9%), Class 2 (intermediate; 31.3%), and Class 3 (high-risk; 32.8%). The high-recovery profile had the highest mean SF-36 mental score (65.1) and autonomy (74.3), the lowest coercion (3.8), the fewest inpatient days (6.3), and the lowest mean direct cost (USD 2621.4). The high-risk profile had the lowest SF-36 mental score (50.8), the lowest autonomy (51.7), the highest coercion (6.9), the most inpatient days (49.4), and the highest mean direct cost (USD 3702.7).

**Figure 2:**
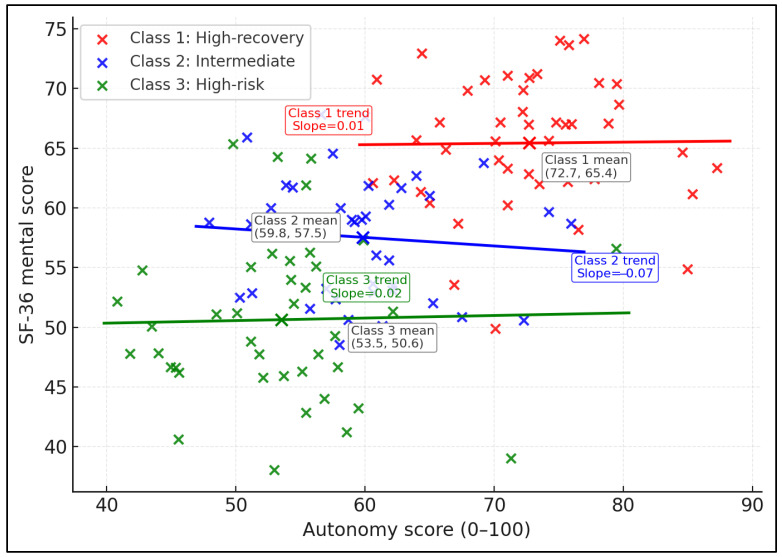
Latent recovery–risk profiles by autonomy and SF-36 mental score.

Figure 2 shows the separation of the three latent profiles in autonomy and SF-36 mental score space using the synthetic coordinates derived from the profile means reported in Table 9.

## 4. Discussion

Our findings show a gradient across Romanian care settings: participants in community protected housing reported the highest mental health-related quality of life and autonomy, the lowest perceived coercion, and the lowest total direct costs; residential homes occupied an intermediate position; and long-stay hospital wards showed the least favorable profile on most outcomes. This pattern is consistent with international supported-accommodation literature showing better autonomy and comparable or lower costs in less institutional settings when community services are adequately organized [16].

The multivariable analyses underline that experiential factors—autonomy and perceived coercion—are more strongly associated with mental health-related quality of life than socio-demographic variables or diagnosis. This is consistent with longitudinal work showing that, over time, subjective quality of life in people with severe mental illness is driven primarily by changes in symptoms, self-perceptions, and social relationships rather than static demographic features [17,18]. Classic reviews have emphasized the role of autonomy, mastery, and self-efficacy as mediators between life circumstances and subjective well-being [17], and a large pooled analysis of over 3900 patients across diagnostic groups found that employment and lower symptom burden were robust correlates of better subjective quality of life, while diagnosis per se explained little variance [19,20]. Our regression model adds to this literature by quantifying the impact of autonomy and perceived coercion on SF-36 mental scores: a 10-point increase in autonomy was associated with nearly a 3-point improvement in mental quality of life, whereas each additional point of perceived coercion corresponded to a measurable decrement. These findings suggest that interventions which explicitly target shared decision-making, procedural justice, and promotion of self-determination may yield benefits that cut across traditional diagnostic boundaries [18,19,20].

Our data also highlight the importance of social resources and meaningful daytime activity. Participation in structured day programs was markedly higher in community protected housing and, in the negative binomial model, was independently associated with roughly 30% fewer inpatient days. Patients in the high-recovery latent profile combined high autonomy and mental quality of life with very frequent day-program participation and family contact, whereas those in the high-risk profile had the weakest social networks and the poorest outcomes. This aligns with studies showing that social network quality and frequency of social interaction are closely linked to both subjective quality of life and broader indicators of recovery in persistent mental illness [19,21]. Eklund and Hansson, for example, found that better quality of life, self-esteem, and more developed social networks were reciprocally related in community samples, implying a dynamic feedback loop between social integration and well-being [21]. Our results suggest that in the Romanian context, day programs and family involvement may be key levers for moving patients from a high-risk to an intermediate or high-recovery trajectory, especially among those already living outside hospital wards.

From a health-economic perspective, the study corroborates the argument that community-based arrangements can be more cost-efficient than prolonged hospital care, even when community support costs are higher. Total direct mental health costs were roughly USD 1000 per patient-year lower in deinstitutionalized settings than in hospital care, driven by large reductions in inpatient bed-days. This finding is consistent with national survey data from England showing that residential care was substantially more expensive than supported housing or floating outreach, without delivering superior quality of life [16]. It also dovetails with the Cochrane review of intensive case management, which demonstrated that well-implemented community-based care can reduce hospital use—especially in populations with high baseline bed use—without worsening symptoms or mortality [22]. Our results differ somewhat in that we observed clear gains in mental health-related quality of life alongside reduced hospitalization, whereas some randomized trials found more equivocal effects on quality of life [22]. One explanation is that we studied “whole-system” differences between sustained institutional and deinstitutionalized pathways, rather than the effect of adding an outreach team to an already community-based system; in settings where hospital-based long-stay care remains prominent, moving patients into protected housing and structured community programs may yield larger marginal benefits.

The latent profile analysis provides a complementary perspective by showing that patterns of autonomy, perceived coercion, service use, and costs cluster into distinct recovery–risk constellations that cut across formal settings. The high-risk profile, characterized by low autonomy, high coercion, heavy inpatient use, and the highest costs, resembles the subgroup of patients with persistent unmet needs and poor social integration described in earlier longitudinal quality-of-life studies [17,20]. Conversely, the high-recovery profile illustrates that even within a resource-constrained system, a sizable minority of patients can achieve good quality of life at relatively low cost when autonomy is high, coercion is low, and community supports are robust. These findings reinforce the view that service planning should not only consider average differences between hospitals, residential homes, and community housing, but also identify and target high-risk profiles wherever they are located. Interventions that strengthen social networks, address depressive and anxiety symptoms, and systematically enhance perceived autonomy are likely to be most impactful for this group [17,19,20,21].

Our findings should be interpreted cautiously. The cross-sectional design precludes temporal ordering, and selection into settings was not random; placement likely reflected clinical severity, functional status, family support, clinician judgment, and local service availability. Accordingly, the observed autonomy and cost gradients cannot be interpreted as causal effects of setting. The exploratory mediation model should also be read as a statistical decomposition of contemporaneous associations rather than proof of a causal pathway.

Within these limits, the overall pattern remained coherent across descriptive, multivariable, and count-model analyses: residential and especially community arrangements outside hospital wards were associated with better patient-reported mental health and lower direct costs. This interpretation is broadly aligned with international evidence on supported accommodation and recovery-oriented care [16,23,24,25,26,27,28,29], but it requires confirmation in longitudinal or quasi-experimental studies. Nevertheless, these findings should be considered in the study context, as different patient factors, regional factors, comorbidities, and hospital setting specific data may influence the applicability of results in different scenarios [30,31,32,33,34,35,36,37,38,39].

This study has several limitations that should be considered when interpreting the results. Its cross-sectional design precludes causal inferences about the effects of setting on outcomes; patients were not randomly allocated to hospital, residential, or community arrangements, so selection effects and residual confounding are likely. The sample was adequate for detecting medium group differences but remained modest for exploratory mediation and latent profile analyses, which should therefore be interpreted cautiously. The sample was drawn from a single Romanian county, which may limit generalizability to other regions or health systems. Autonomy and perceived coercion were assessed using brief study-specific items rather than full-length instruments such as the MacArthur Admission Experience Survey, potentially undercapturing nuances of procedural justice and perceived pressure. Cost estimates were restricted to direct mental health expenditures and did not include indirect costs, wider social care spending, employment effects, or caregiver burden.

## 5. Conclusions

Among the three Romanian care settings compared in this study, community protected housing showed the most favorable profile, residential care occupied an intermediate position, and hospital care showed the lowest autonomy and the highest direct costs. When residential and community services were combined as deinstitutionalized arrangements, these settings were associated with better mental health-related quality of life and lower direct costs than long-stay hospital care. Across all settings, autonomy and perceived coercion were more strongly associated with mental health-related quality of life than diagnosis or basic socio-demographic characteristics. These cross-sectional findings support further expansion of recovery-oriented residential and community services in Romania, but longitudinal and interventional studies are needed before causal conclusions can be drawn.

## Figures and Tables

**Table 1:** Socio-demographic and clinical characteristics by care setting.

Variable	Hospital (*n* = 42)	Residential (*n* = 43)	Community (*n* = 43)
Age, years (mean ± SD)	47.7 ± 10.6	53.8 ± 8.8	45.1 ± 11.2
Female, %	54.8	58.1	37.2
Education, years (mean ± SD)	11.2 ± 1.9	9.8 ± 2.0	11.4 ± 2.1
Low income, %	73.8	65.1	58.1
Illness duration, years (mean ± SD)	12.5 ± 5.2	14.1 ± 6.1	10.7 ± 4.1
DSM-5 psychotic disorder, %	45.2	62.8	44.2

SD—standard deviation; *n*—number; %—percentage. Note: Diagnoses were coded according to DSM-5 categories; psychotic disorder refers to schizophrenia spectrum and schizoaffective disorders.

**Table 2:** Institutional trajectory and service configuration.

Variable	Hospital (*n* = 42)	Residential (*n* = 43)	Community (*n* = 43)
Prior psychiatric hospitalizations, *n* (mean ± SD)	7.4 ± 2.7	5.6 ± 3.8	4.4 ± 3.0
Years in current setting (mean ± SD)	5.1 ± 2.7	7.1 ± 3.5	2.9 ± 1.9
Participation in day programs, %	52.4	58.1	81.4
Weekly family contact, %	42.9	48.8	67.4

SD—standard deviation; *n*—number; %—percentage.

**Table 3:** Quality of life, autonomy, and perceived coercion.

Variable	Hospital (*n* = 42)	Residential (*n* = 43)	Community (*n* = 43)
SF-36 physical score (mean ± SD)	57.1 ± 8.7	58.0 ± 7.9	61.9 ± 7.7
SF-36 mental score (mean ± SD)	52.8 ± 9.1	56.0 ± 8.6	63.3 ± 7.6
Autonomy score (0–100, mean ± SD)	53.0 ± 8.6	57.7 ± 9.5	72.1 ± 10.0
Perceived coercion (0–10, mean ± SD)	6.4 ± 1.5	6.1 ± 1.6	4.2 ± 1.3

SF-36—36-Item Short Form Health Survey; SD—standard deviation.

**Table 4:** Annual service use and direct mental health costs.

Variable	Hospital (*n* = 42)	Residential (*n* = 43)	Community (*n* = 43)
Inpatient days/year (mean ± SD)	47.5 ± 17.7	17.0 ± 9.9	7.8 ± 6.4
Outpatient visits/year (mean ± SD)	5.5 ± 2.7	8.2 ± 3.3	12.4 ± 3.8
Medication cost/year, USD (mean ± SD)	1008.9 ± 177.0	1021.5 ± 184.4	1043.3 ± 192.0
Community support cost/year, USD (mean ± SD)	384.5 ± 113.5	685.3 ± 169.5	957.8 ± 150.0
Total direct cost/year, USD (mean ± SD)	3666.2 ± 820.7	2679.5 ± 480.8	2663.8 ± 372.9

SD—standard deviation; USD—United States dollars. Note: Original mean costs in RON were approximately 4641, 4699, and 4799 for medication costs; 1769, 3152, and 4406 for community support costs; and 16,865, 12,326, and 12,254 for total direct costs in hospital, residential, and community settings, respectively. USD equivalents were derived using the mean 2024 NBR exchange rate (approximately 1 USD = 4.6 RON).

**Table 5:** Clinical outcomes and costs by deinstitutionalization status.

Variable	Hospital (*n* = 42)	Deinstitutionalized (*n* = 86)	*p*-Value
Readmission within 12 months, %	47.6	29.1	0.062
Emergency department visits, %	40.5	31.4	0.41
Coercive measures, %	26.2	19.8	0.55
SF-36 mental score (mean ± SD)	52.8 ± 9.1	59.7 ± 8.9	<0.001
Total direct cost/year, USD (mean ± SD)	3666.2 ± 820.7	2671.6 ± 427.8	<0.001

Note: The mean total direct cost in the deinstitutionalized group corresponded to approximately 12,289 RON/year. SF-36 = 36-Item Short Form Health Survey; SD = standard deviation; USD = United States dollars.

**Table 6:** Multivariable linear regression predicting SF-36 mental score.

Predictor	B (Unstandardized SF-36 Points)	SE	*p*-Value
Age (years)	0.05	0.07	0.51
Female (vs. male)	1.99	1.48	0.18
Low income	–0.55	1.57	0.73
Psychotic diagnosis (vs. others)	2.56	1.49	0.09
Deinstitutionalized setting	1.65	1.77	0.35
Autonomy (per 10-point increase)	2.79	0.7	<0.001
Perceived coercion (per 1-point increase)	–1.32	0.48	0.007

Model R^2^ = 0.316; F(7, 120) = 7.91; *p* < 0.001; SF-36—36-Item Short Form Health Survey; B—unstandardized regression coefficient; SE—standard error; R^2^—coefficient of determination; F—F statistic; *p*—*p*-value. Note: Unstandardized coefficients are shown to preserve direct interpretability in SF-36 points.

**Table 7:** Negative binomial regression for annual psychiatric inpatient days.

Predictor	Incidence Rate Ratio (IRR)	95% CI for IRR	*p*-Value
Deinstitutionalized setting (vs. hospital)	0.3	0.2–0.4	<0.001
Autonomy (per 10-point increase)	0.8	0.7–0.9	0.004
Perceived coercion (per 1-point increase)	1.1	1.0–1.3	0.043
Day-program participation (yes vs. no)	0.7	0.6–0.9	0.006
Psychotic disorder (yes vs. other diagnoses)	1.2	0.9–1.5	0.173
Age (per 10-year increase)	1	0.9–1.1	0.827

Outcome: number of inpatient days in the last 12 months (negative binomial model); model adjusted for sex and low-income status. IRR < 1.0 indicates fewer inpatient days; IRR—incidence rate ratio; CI—confidence interval; *p*—*p*-value.

**Table 8:** Mediation Analysis: Autonomy as a mediator between deinstitutionalized setting and SF-36 mental score.

Path/Effect	Unstandardized Effect (B)	SE	95% CI	*p*-Value
(a) Setting → Autonomy	11.4	1.8	7.9–14.9	<0.001
(b) Autonomy → SF-36 mental (per 1-point increase)	0.3	0.1	0.1–0.5	0.003
(c) Total effect: Setting → SF-36 mental	6.9	1.7	3.5–10.3	<0.001
(c′) Direct effect: Setting → SF-36 mental (adj. for autonomy)	3.4	1.6	0.3–6.5	0.034
(ab) Indirect effect via autonomy	3.5	—	1.6–5.4	0.001

Predictor: deinstitutionalized setting (residential or community = 1, hospital = 0). Mediator: autonomy score (0–100). Outcome: SF-36 mental component score; SF-36—36-Item Short Form Health Survey; B—unstandardized regression coefficient; SE—standard error; CI—confidence interval; *p*—*p*-value.

**Table 9:** Latent profile analysis of recovery and risk patterns.

Variable	Class 1—High-Recovery Profile (*n* = 46)	Class 2—Intermediate Profile (*n* = 40)	Class 3—High-Risk Profile (*n* = 42)
Class size, % of sample	35.9	31.3	32.8
SF-36 mental score (mean)	65.1	57.4	50.8
Autonomy (0–100, mean)	74.3	60.2	51.7
Perceived coercion (0–10, mean)	3.8	6.1	6.9
Inpatient days/year (mean)	6.3	18.1	49.4
Outpatient visits/year (mean)	13.7	8.6	5.3
Day-program participation, %	87	61.5	39.7
Weekly family contact, %	78.3	55.2	28.6
Total direct cost/year, USD (mean)	2621.4	2768.9	3702.7

Three latent classes were derived from SF-36 mental score, autonomy, perceived coercion, inpatient days, outpatient visits, and costs using latent profile analysis; class enumeration selected using lowest Bayesian Information Criterion and acceptable entropy; differences across classes for continuous indicators were significant at *p* < 0.001 (ANOVA); SF-36—36-Item Short Form Health Survey; USD—United States dollars; ANOVA—analysis of variance; *p*—*p*-value.

## Data Availability

The data are not publicly available due to privacy and ethical restrictions. Data availability is subject to institutional and hospital approval.
